# From digital health to learning health systems: four approaches to using data for digital health design

**DOI:** 10.1080/20476965.2023.2284712

**Published:** 2024-01-13

**Authors:** Valeria Pannunzio, Maaike Kleinsmann, Dirk Snelders, Jeroen Raijmakers

**Affiliations:** a Department of Design, Organization and Strategy, Faculty of Industrial Design Engineering, Delft University of Technology, Delft, the Netherlands; b Philips Experience Design, Philips, Eindhoven, the Netherlands

**Keywords:** Healthcare design science, clinical systems and Informatics, decision support systems, data mining & data analytics

## Abstract

Digital health technologies, powered by digital data, provide an opportunity to improve the efficacy and efficiency of health systems at large. However, little is known about different approaches to the use of data for digital health design, or about their possible relations to system-level dynamics. In this contribution, we identify four existing approaches to the use of data for digital health design, namely the silent, the overt, the data-enabled, and the convergent. After characterising the approaches, we provide real-life examples of each. Furthermore, we compare the approaches in terms of selected desirable characteristics of the design process, highlighting relative advantages and disadvantages. Finally, we reflect on the system-level relevance of the differentiation between the approaches and point towards future research directions. Overall, the contribution provides researchers and practitioners with a broad conceptual framework to examine data-related challenges and opportunities in digital health design.

## 1. Introduction

### 1.1. The digital health revolution(s) and the role of design

Health systems worldwide face widespread challenges. Long-term demographic and epidemiological trends, combined with new, disruptive phenomena such as the COVID-19 pandemic, result in a worrisome combination of systemic understaffing (Drennan & Ross, 2019) and increasing costs of care (Chang et al., 2019). One of the directions undertaken to relieve health systems from these pressing issues is the incremental adoption of digital technologies in the health domain, often referred to as the digital health revolution (see e.g., Powell & Arvanitis, 2015; Snyder & Zhou, 2019).

This revolution, described as ongoing, has undergone several phases; from the introduction of ‘health telematics’ in the 1970s, to the diffusion of the Internet and the Personal Computer in the 21st century, to the advent of mobile health technologies in the 2010s (Manteghinejad & Javanmard, 2021). A contemporary frontier of the digital health revolution is represented by the growing use of AI and ‘smart’ technologies in the health domain, comprehensively described by Rajpurkar et al. (2022).

Throughout its different waves, the digital health revolution has been supported by digital health design, intended as the processes required for designing new digital health artefacts or redesigning existing ones. Digital health design processes typically include different phases, including research, development and testing; and can involve patient input and participation to varying degrees (Birnbaum et al., 2015).

### 1.2. Digital health design and health systems

For digital health artefacts to deliver their much-needed benefits in clinical practice, they must be designed to be not only effective and safe, but also to seamlessly integrate within existing health systems. In turn, on an aggregated level, the way digital health artefacts are designed affects health systems’ characteristics and functioning, contributing to their complex and adaptive behaviour (Rouse, 2008).

To manage and optimise health systems’ complex adaptive behaviour, the notion of the Learning Health System (LHS) has been formulated as a desirable paradigm of digitally enabled, continuous healthcare improvement (Friedman et al., 2010). In LHSs, routine healthcare data such as that which is collected in electronic health records (EHRs) is used for continuous learning and improvement purposes, including health innovation development and testing. While recent studies indicate that much work must be conducted before the vision of a functioning, efficient LHS at a national scale is realised in practice (Zhang et al., 2023), LHSs remain a useful vision to tend towards in the digital health domain.

This has implication for the field of design: Greene et al. (2012), for instance, propose the embedding of participatory design processes within LHSs continuous improvement cycles, noting that LHSs could generate useful information on design problems and new solutions. Furthermore, LHSs would rely on data interfaces embedded in digital health design artefacts, which would ideally be designed for this purpose. Yet, little research to date has explored the links between LHSs and design practice, such as the potential of design processes to support the transition towards LHSs or the need to adapt existing design approaches in LHS scenarios. Therefore, at this stage, it is unclear how design processes could fit within and contribute to the continuous streams of data generated in LHSs. We characterise this as a relevant research gap in our pivotal stage of digital health innovation, and note that, as we increasingly move towards AI-driven, smart health solutions, it is crucial to critically examine the role played by data within digital health design processes and its system-level implications.

In the present contribution, we explore this topic by proposing a first broad conceptual distinction among existing approaches to the use of data in digital health design. To do so, we build on design, digital innovation, and digital health literature on one side, and the authors’ first-hand experience in digital health practice on the other side. This distinction between approaches is meant to support practitioners and researchers who wish to examine data-related system-level challenges and opportunities while navigating the landscape of existing digital health design practices.

The paper is set up as follows. First, we briefly elaborate on key concepts such as digital health design and data. Secondly, we introduce a proposed distinction into four existing approaches to the use of data in digital health design processes. Following, each approach is examined in terms of use of data for design decision-making, and exemplified through a brief real-world case description. Finally, the four approaches are compared and future research directions are outlined.

## 2. Digital health design and data

### 2.1. Designing digital health: scope and state of the art

Designing digital health artefacts involves a number of possible sub-processes articulated across different stages, including empathising, prototyping, gathering user feedback, pilot testing, evaluating, and more (Mummah et al., 2016). In this contribution, we strive to consider design processes across their full spectrum of activities, since each phase – from early-stage to post-implementation – is relevant to the question of data practices. For the same reason, we adopt a broad definition of digital health, in accordance with the characterisation offered by the U.S. Food and Drug Administration (2020). Therefore, we consider a broad range of digital health artefacts including products with digital capabilities (e.g. sensors and wearables), digital-only propositions (such as health apps or digital platforms), as well as complex, hybrid interventions (e.g. remote patient monitoring services).

Previous research has investigated this diverse, fast-developing field, distilling precious knowledge on digital health design theory and practice. Particularly, it has been noted that the complex, interdisciplinary nature of the digital health domain determines unique challenges for the design function (Duffy et al., 2022; Pagliari, 2007), challenging traditional design principles (van Velsen et al., 2022) and requiring new, ad-hoc frameworks (Kowatsch et al., 2019) and approaches (Van Velsen et al., 2013). While the field has experienced substantial growth and progress in recent years, it is still characterised by a fast-evolving landscape, with a growing body of evidence, methodologies, and technical innovations shaping its trajectory.

### 2.2. Data: definitions and role in design processes

The concept of ‘data’ is a broad one, rich of different interpretations and uses (Furner, 2016). Traditionally, data is intended in abstract as ‘symbols that represent the properties of objects and events’, which can be progressively processed and distilled into information, knowledge and wisdom (Ackoff, 1989). In the digital world, data are often intended as electronically stored and processed; however, in this contribution we will consider both digital data and analog data, typically found in the physical world.

Throughout the developments affecting the digital health domain, a common thread is represented by the increasing importance of data as the fuel of digital health transformation (Haggerty, 2017). Data access is, in fact, not only crucial for the continuous functioning of existing digital health interventions, but also for digital health innovation (Gopal et al., 2019), including the process of designing new digital health artefacts or redesigning existing ones. Recently, the role of data within design processes has been a popular subject of investigation in design literature (Cantamessa et al., 2020). Through these efforts, relevant conceptual progress has been achieved. Particularly, Wolff et al. (2016) distinguish between ‘designing from data’, intended as the use of data within the design process for inspiration, evaluation of other purposes, and ‘designing with data’, intended as the use of data as design material through its integration in the designed artefacts, as in the case of smart devices or systems ^1^.

A related distinction is operated by Briard et al. (2023), who differentiate between ‘external data’ and ‘captured data’ as input for product design processes – the latter referring to data that originates from the product itself (e.g., through built-in sensors or interface usage monitoring). In the following section, we apply these insights and findings to the specificities of the digital health domain, in which we distinguish a range of existing data-related approaches.

## 3. Four approaches to using data for digital health design

**Figure 1.**
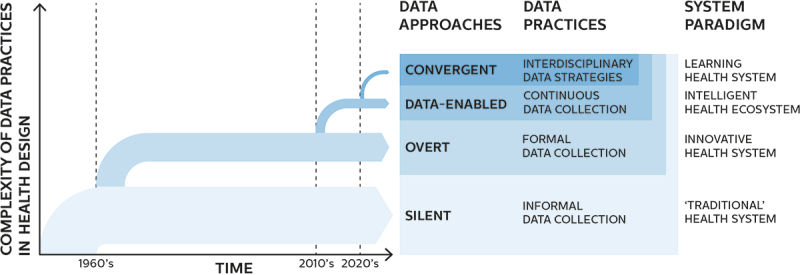
Approaches to using data in digital health design ordered by time and complexity of data practices.

To articulate our differentiation between approaches to the use of data in digital health design, we proceed along time and complexity dimensions. Specifically, we start from the oldest and simplest possible form of data use and incrementally transition towards more novel, complex, and professionalised practices, highlighting key developments and health systems implications along the way. A summarising overview is provided in Figure 1.

The first approach we characterise, the *silent*, stems from a recognition that not all design activities in the health domain are explicitly recognised as design. Since our scope is to consider the broad landscape of real-world health design practice and its impact on health systems, it is important for us to include design activities that might not be explicitly recognised as design while effectively fulfilling the design function. We are helped in this by traditional design literature, and more precisely by Gorb and Dumas (1987) who first introduced the notion of *silent* design as ‘design by people who are not designers and are not aware that they are participating in design activity’. Expressed in these terms, *silent* design appears to constitute a common occurrence in the history of healthcare, in which design activities have been conducted long before the formalisation and professionalisation of design disciplines. To this day, countless new health solutions, including digital ones, keep on being developed without the involvement of professional designers. Often, the ‘*silent’*, non-professional health designers are individuals or groups who are invested in the context of the innovation, be it as healthcare professionals, as patients, or as patients’ loved ones. Since silent designers do not follow formal design processes, their data practices – if present at all – tend to be mostly informal and unstructured, e.g., taking the form of brief searches to aid prototyping or spontaneous inquiries to validate early ideas. Therefore, we borrow the term *silent* to describe the simplest possible approach to the use of data for health design, in which a structured design process is not followed and data practices are informally conducted (if present at all). While this first approach is relatively unsophisticated, it can support the provision of the design function within “traditional” health systems, as it has long been the case before the advent of professional designers.

The seminal Gorb and Dumas paper from 1987 introducing the concept of *silent* design also offers a designation of its opposite: *overt* design, intended as design conducted by professionally trained designers who knowingly and purposefully engage in design activities. Unlike silent designers, overt designers tend to follow structured design processes, which do incorporate data practices involving formalised data collection, e.g., in the form of generative research (Sanders & Stappers, 2012) or usability testing (Bastien, 2010). We thus borrow the term *overt* to describe a second approach to digital health design, in which structured design processes are conducted and include formalised data collection practices. While examples of overt design in the healthcare domain can be traced back to the 1960s with Bruce Archer’s hospital bed (Archer, 1964), professional health design has since developed in both scope and ambition (see Chamberlain & Craig, 2017; Park, 2015 for useful overviews). Today, overt design approaches support a paradigm of professionalised, structural innovation within health systems, while not replacing silent design practices which continue running in parallel.

In addition to *silent* and *overt* approaches, which are applicable, but not specific to the design of digital health artefacts, we note that unique characteristics of the digital health domain determine the emergence of novel, dedicated design approaches. In particular, two characteristics of digital health artefacts challenge traditional ways of designing. These are:

- The capacity of digital health artefacts to perform based on digital data, including data collected and analysed in real-time;
- The capacity of digital health artefacts to change and evolve over time, including while in use.

These characteristics are enabled by fundamental properties of digital technologies, namely data homogenisation and re-programmability (Yoo et al., 2012). Data homogenisation refers to the capacity of all digital data to be ultimately converted in binary numbers, while re-programmability refers to the capacity of digital devices to perform a wide array of functions through their flexible architecture.

*Data-enabled* design approaches deal with these specificities through the establishment of continuous (re)design loops informed by data collected by = digital health artefacts in the context - which Briard et al. (2023) call captured data. This principle is currently applied in many non-health-related sectors, such as entertainment, transportation, or retail, in which usage data is routinely employed to gain inspiration for new service features, to develop them, to test them, to update them, and more. In the field of digital health, the possibility and usefulness of establishing closed loops of data continuously informing design processes have been demonstrated in the last decade by Van Kollenburg and Bogers (2019) through their extensive work on data-enabled design in the health domain. An interesting implication of these developments for design theory is the changing role of the designed artefact itself, which becomes not only an output of the design process, but also a source of continuous information through the establishment of a “built-in” data infrastructure.

As a consequence, this data infrastructure has to be designed as part of the artefact itself: the supported health system paradigm is in this case the one of the *intelligent ecosystem*, intended by Van Kollenburg and Bogers (2019) as a dynamic composition of interrelated products, services and people which can use data and artificial intelligence to learn about users and contexts.

In consideration of these elements of novelty in design processes following *data-enabled* approaches, we find it appropriate to differentiate these from *overt* approaches, even though *data-enabled* approaches emerge from and build upon traditional, non-data-enabled design theories and methodologies.

The capacity of digital health artefacts to change and evolve over time determines the need for continuous and contextual post-adoption evaluation. A concrete example in the field of digital health can be found in a recent study regarding a widely adopted proprietary sepsis prediction model, which unexpectedly revealed concerning underperformance at a large scale (Wong et al., 2021). In response to this need, continuous data collection from the context of use becomes necessary to inform processes other than design-related ones: in the healthcare domain, particularly, clinical evaluation, clinical research and auditing programs require dedicated data infrastructures supported by a network of compatible digital artefacts. In this context, a fourth kind of approach to using data for digital health design emerges, in which shared data strategies (Pannunzio et al., 2023b) need to be employed both for design purposes and for other kinds of health-relevant data-driven processes, such as clinical evaluation, cost evaluation, policymaking, algorithmic auditing, or more. The establishment of these shared data strategies (intended as the embedding of data collection capabilities for a diverse set of purposes within digital health artefacts) is identified as a new layer of complexity on top of data-enabled design approaches, leading us to distinguish these as a new set of emerging new approaches. We refer to these approaches as *convergent*, in association with the concept of digital convergence as intended by Yoo et al. (2010) and of convergence as the ‘integration of insights and approaches from historically distinct scientific and technological disciplines’ in health innovation as intended by Sharp et al. (2016). While examples of convergent approaches appear at this stage to be rare in the digital health innovation landscape, we note that they might be instrumental in supporting the LHS paradigm, at least in the measure in which they could facilitate the capturing of new, interdisciplinary knowledge ‘as an integral by-product of the delivery experience’ (McGinnis et al., 2013).

Overall, we remark that the described approaches are incremental rather than mutually exclusive; in other words, new data practices are added at each step, rather than substituted. While other approaches could be described and other ways of segmenting the landscape of health digital design practice could certainly be proposed, we choose this particular demarcation since it highlights developments that are directly relevant to the issue of health systems digitisation.

Next, we will further examine the four approaches by focusing on *design decision-making* as a mechanism through which the design function affects the final characteristics of digital health artefacts. This characterisation draws from Simon’s seminal work on decision-making (Simon, 1977), reflected both in traditional software design literature (including Freeman & Wasserman, 1983, who remark that ‘decision-making is what design is all about’), in engineering design literature (Badke-Schaub & Gehrlicher, 2003), and in related biomedical informatics literature (Jalote-Parmar et al., 2010). In particular, we will examine the way data is collected and used for design decision-making, intended as the broad variety of data used to gain inspiration, formulate hypotheses, test assumptions, or evaluate solutions as part of digital health design processes. Particularly, we will consider the way data is collected from (parts of) the underlying health system, and the way data is used to make design decisions about digital health artefacts. Furthermore, we will offer real-life examples of each process. We note that these examples are meant to illustrate real-world occurrences of the four approaches, rather than provide an exact delineation between the described data practices.

### 3.1. Silent approaches to digital health design

**Figure 2.**
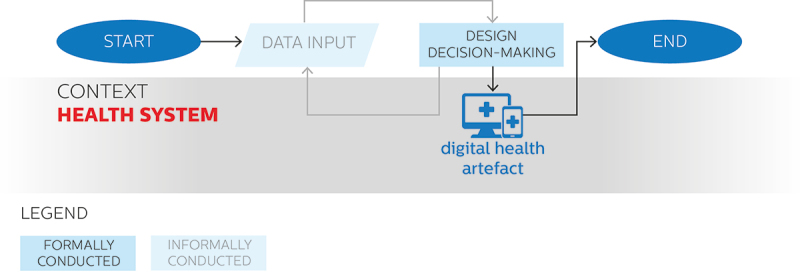
Use of data in silent approaches to digital health design depicted on a flowchart diagram.

We have previously mentioned that silent approaches to the use of data for digital health design are often adopted by patients, health professionals, or other individuals or groups holding a direct stake in the context of application, who do not necessarily follow a structured design process. In these approaches, data collection for design decision-making tends to be skipped – or, perhaps more precisely, to be carried out implicitly. This is because, due to the familiarity of silent designers with the context and the design problem at hand, design decision-making can happen naturally and intuitively: in a way, necessary data are already implicitly in possession of the silent designer, and as such do not need to be explicitly collected nor formally analysed. However, design decision-making can still proceed iteratively, as in the example provided in the next section. In any case, the outcome of the design process is a digital health artefact, which if successfully implemented and adopted becomes a part of the healthcare system. Figure 2 provides an overview of the characteristics of the use of data in silent approaches to digital health design depicted as a flowchart diagram.

### 3.2. Example: the do-it-yourself artificial pancreas system (DIYAPS)

Dana Lewis, a person with diabetes ‘with no medical or technology background whatsoever’ (Lewis, 2019) is a creator of the Do-It-Yourself Artificial Pancreas System, a widely adopted hybrid closed loop system to automate micro-adjustments of insulin delivery based on real-time glucose monitor data. Lewis describes the process leading up to the development of the system in detail in a dedicated blog (Lewis, 2016), from the initial frustration with existing medical devices, to the series of incremental self-experimentations and improvements eventually leading her to obtain a functioning closed-loop system, to the decision of sharing her knowledge publicly in an open-source format, enabling other patients to build their own systems. Today, thousands of individuals have reportedly implemented various kinds of DIY closed-loop solutions based on Lewis’ work in their own everyday diabetes care routines (OpenAPS Outcomes, 2021).

Lewis describes her design decision-making process to be intuitive and spontaneous: ‘at every stage, it was very easy to see what I wanted to do next and how to iterate, despite the fact that I am not a designer and I am not a traditional engineer’ (Lewis, 2016). It could be argued that, in her case, being a formally trained designer was not required, since she already possessed (through first-hand, real-life experience) the information necessary to conceptualise and definewhat would constitute a desirable digital health solution. Following its widespread adoption, the Do-It-Yourself Artificial Pancreas System has been evaluated in formal studies (Jennings & Hussain, 2020), reporting tangible benefits, including decreased HbA1c values and increased TIR (time in range). Currently, the system is being further developed and evaluated in the OPEN study, an initiative funded by the European Commission’s Horizon 2020 Research and Innovation Program (O’Donnell et al., 2019).

### 3.3. Overt approaches to the use of data for digital health design

**Figure 3.**
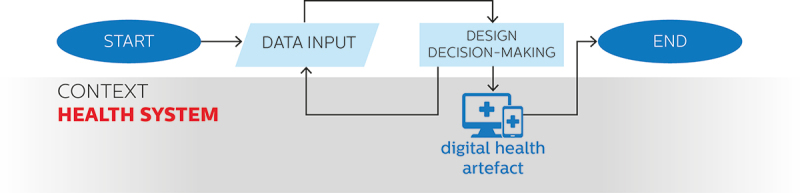
Use of data in overt approaches to digital health design depicted on a flowchart diagram.

We have previously introduced the overt as a second approach to the use of data for digital health design, conducted by professional designers who do follow formal design processes – a relatively common occurrence in the modern health tech sector, in which the design function is increasingly professionalised. In overt approaches, design decision-making is thus formally conducted and informed by purposefully collected data, usually in an iterative fashion. Figure 3 provides an overview of the characteristics of the use of data in overt approaches to digital health design depicted as a flowchart diagram.

### 3.4. Example: the computerised AKI (acute kidney injury) decision support tool

Acute kidney injury (AKI), previously known as acute renal failure, is the clinical manifestation of a diverse set of disorders affecting the kidney acutely (Bellomo et al., 2012). Such clinical manifestation is particularly common among the critically ill, and it has been reported to occur in more than half of patients at some point of a critical care admission (Hoste et al., 2015). In 2016, a project was initiated as an internal collaboration between Philips Research North America and Philips Design, with the aim of improving early recognition and management of AKI in intensive care units through automated electronic alerts coupled with a clinical decision support system (CDSS). Professionally trained designers worked on transforming this idea into an implementable service solution. As part of the design process, qualitative data was purposefully collected, through dedicated interviews and workshops, on aspects such as intensive care nurses’ and clinicians’ experiences and preferences with regard to clinical decision support systems. The design process resulted in a set of recommendations for the development of the service. A prototype system developed in co-creation with the clinical team at University Hospital Bristol was later tested in a prospective observational study, which reported a relation between the adoption of the system and a decrease in the proportion of patient worsening from stage 1 AKI, a decrease in the proportion of incorrect enoxaparin dosage, and a decrease in the overall prevalence of any AKI in the involved intensive care units (Bourdeaux et al., 2020).

### 3.5. Data-enabled approaches to digital health design

An opportunity enabled by the formalisation of design decision-making processes in the field of digital health is the establishment of continuous loops of redesign, informed by data collected directly by the digital health artefact while in use. Data-enabled approaches seize this opportunity by purposefully designing built-in infrastructures for the continuous collection of contextual data as part of the digital health artefact itself. This happens in addition rather than in substitution of data practices adopted in overt design approaches, increasing the complexity involved in the design process.

**Figure 4.**
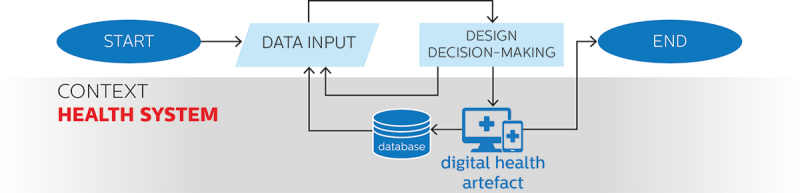
Use of data in data-enabled approaches to digital health design depicted on a flowchart diagram.

Figure 4 provides an overview of the characteristics of the use of data in data-enabled approaches to digital health design depicted as a flowchart diagram.

### 3.6. Example: the co-responsibility study

The co-responsibility study is a research and design project following a data-enabled approach (Jansen et al., 2020). The project focused on the design of an open system to support health behavioural change after bariatric surgery. The system was meant to connect patients, partners, healthcare professionals, and involved researchers, and was devised to include data from different sources including medical records, self-reported data, and contextual data. Its functionalities were not pre-set, and could be modified remotely during the study itself.

The study participants themselves could reflect on the collected data and were actively engaged in the research. Through the study and its data, design-relevant use cases were found that could bring value to the system users, including ideas for new functionalities. Furthermore, the data-enabled nature of the study allowed the design team to reach a deeper, more nuanced understanding of the complex dynamics underlying the relationships between patients, their partners, their health professionals, and of how these dynamics contribute to shaping everyday life health behaviour.

The management of the collected data in the study was carefully orchestrated by a core design team, including design researchers, data designers, and service designers, who collectively shaped the system data infrastructure through interactive dashboards (Lovei et al., 2020) and visual system maps (Pannunzio et al., 2020). In 2021, a concept called CoreCare, originated from the Co-responsibility study, was awarded a Red Dot Design Concept award (Dot, 2021).

### 3.7. Convergent approaches to digital health design

**Figure 5.**
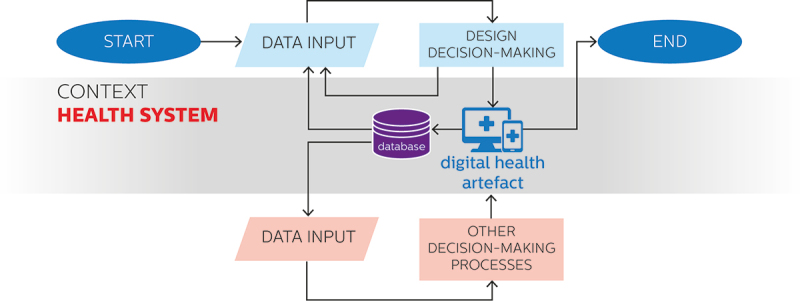
Use of data in convergent approaches to digital health design depicted on a flowchart diagram.

The creation of infrastructures dedicated to collecting real-world data about the functioning of digital health solutions unlocks the chance to employ the continuously collected data for decision-making processes other than design-related ones, such as clinical evaluation, cost evaluation, policymaking, algorithmic auditing, or more. Convergent approaches seize this opportunity through the development of interdisciplinary, shared data strategies devised to inform both design decision-making and other relevant data-driven processes, such as clinical evaluation, cost evaluation, policymaking, algorithmic auditing, or more. Once again, these kinds of data practices build on and add to the ones adopted in data-enabled approaches, determining an increase in process complexity. Figure 5 provides a depiction of the characteristics of the use of data in convergent approaches to digital health design depicted on a flowchart diagram.

### 3.8. Example: the perioperative box

Major gastrointestinal surgeries are associated with a relatively high incidence of postoperative complications (Jakobson et al., 2014). In 2019, a multi-organisation collaboration led by the Leiden University Medical Center in the Netherlands was initiated to develop and test a system for continuous remote monitoring and early diagnosis of complications following major gastrointestinal surgeries. The system is meant to be a digital health artefact involving a complex set of interconnected monitoring devices, actors and interfaces, including a machine learning algorithm generating alarms to flag patients at risk of developing complications. Separate data collection activities have been carried out by the different actors involved in the design and evaluation of the system at different points; in addition, a research protocol for a pilot study to assess the feasibility of the intervention has been developed by an interdisciplinary team, including medical, technical and design experts. The protocol includes the collection of data necessary for the assessment of assumptions related to the design of the digital health proposition, and also the collection of data necessary for the assessment of clinically relevant assumptions, such as the predictive value of the self-monitored data. Importantly, these two sets of data are not only collected from the same context, but also materially overlap. This is the case, for example, of data on patients’ compliance rates to the self-monitoring protocol, including the use of a blood pressure cuff, a smartwatch and a smart thermometer.These data sets are, on one side, instrumental for reaching an understanding of the intervention’s potential in terms of predictive capacity;on the other side, they can inform future redesigns of the suggested self-monitoring routine itself, or of the informational material included as part of the intervention.

## 4. Comparing the four approaches

As demonstrated by the provided examples, each one of the described approaches can result in the development of new, valuable digital health artefacts. While it is impossible at this stage to impart any value judgement on the overall merit of any approach over the other, we believe each to have unique advantages in terms of desirable characteristics of the design process.

In particular, we note how silent approaches possess, more than others, the desirable characteristic of *context embeddedness*, intended as an intimate understanding of the new artefact’s context of application, afforded by a personal familiarity with it. While this understanding might have its limitations (such as it being based on an individual perspective rather than on the experiences of a broader group), it must be noted that it occurs before the start of the design process itself. In overt design approaches, context embeddedness is often pursued explicitly, through methodological traditions such as user-centred (Abras et al., 2004) and human-centred (Gulliksen et al., 2003) design, including a broad spectrum of participatory and co-design practices (Sanders & Stappers, 2008). ‘Good’, successful user-centred design processes can and do achieve considerable results in terms of context embeddedness. However, it must be noted that user-centred techniques are useful precisely because familiarity with the context and understanding of the user perspective are not the starting condition of the overt design process. As such, overt design approaches always run a risk of context detachment compared to silent approaches, and this risk requires mitigation through high-quality design practice.

On the other hand, overt approaches possess the desirable characteristic of *formalisation*, since the professionalisation of design practice confers an improved degree of accountability and communicability to the design function. The development of specialised knowledge dedicated to the disciplinary field of design undertaken across the past decades has detailed and expanded the formalisation of design practice, equipping professional designers with practical and theoretical resources to manage the design process without endangering the creativity of outputs from that process. Younger branches of design practice, such as data-enabled approaches, have more recently undertaken a process of formalisation noticeable in published literature (Bogers et al., 2016, 2016, 2018, 2018; Gulotta et al., 2013; Jansen et al., 2020; Lovei et al., 2020; Van Kollenburg et al., 2018; Yang et al., 2018); while convergent approaches have only been conceptualised in the past few years (Pannunzio et al., 2019; 2023b; Sharp et al., 2016).

A consequence of the formalisation of design practice is the purposeful establishment of flows of information, collected through heterogeneous data sources, to support design processes. As design projects gain complexity, increase in sample sizes, and move into the digital realm, effective *data management* emerges as a desirable characteristic of design processes. In advanced examples of effective data management within design processes, such as data-enabled design projects, digital data becomes a material that can effectively fuel continuous loops of improvement. At least in principle, therefore, data-enabled design approaches can offer, more than others, the advantage of *data management* within design processes.

Finally, as design practice becomes a continuous, data-driven process and as it expands in domains dominated by other data-driven sources of decision-making, the practical need emerges for design to interface with data-driven processes from other disciplines, most prominently through data sharing and interdisciplinary analysis. In the case of healthcare, crucial decision-making related to the adoption of new artefacts largely depends on evidence-based clinical research processes belonging to the well-established disciplinary realm of medical sciences. In these cases, *data sharing* becomes a desirable characteristic of design processes, at least in the measure in which the sharing of data between design and other decision-making processes is beneficial for the larger innovation process. At the same time, data-driven interdisciplinary collaboration can be instrumental in challenging, nuancing, enriching and complementing traditional clinical research methodologies, especially in areas in which these might be less effective in terms of accurately capturing and describing complex phenomena (such as heterogeneous pathophysiologies, context-dependent sociotechnical interventions, or multifarious outcome measures).^2^ At least in principle, convergent approaches can facilitate and support mechanisms of *data sharing* as part of design processes. Table 1 summarises and compares the strengths of the four approaches. It can be noticed from this comparison that each approach unlocks the possibility for the next; e.g., without some degree of formalisation in the design process, it would be impossible to conceptualise a systematic use of data for design purposes, and so on for each step.

**Table 1.** Relative strengths of the four approaches to the use of data for digital health design on four desirable characteristics of the design process. Cells with question marks indicate the described strength to be still hypothetical, rather than robustly observed.

	Silent approaches	Overt approaches	Data-enabled approaches	Convergent approaches
Context embeddedness	++	+	+	+
Formalisation	n/a	++	+	?
Data management	n/a	n/a	++?	+?
Data sharing	n/a	n/a	n/a	++?

Each novel approach constitutes an incremental improvement from the point of view of a desirable characteristic of the design process, but the improvement tends to come at the cost of reduced control on antecedent desirable characteristics. In this perspective, the four approaches might be described as a reflection of the growing sophistication of design as a field. While silent design might perfectly meet the needs of the single user or small group of users involved in the process, it may lack the wider stakeholder engagement that overt approaches support. In turn, data-enabled approaches expand the multiple stakeholders’ perspective with contextual data accumulated as part of the design process, while convergent approaches allow for data-driven learning across and between different fields and perspectives. These changes correspond to an evolution of the role of the professional designer, as a figure who draws from an increasingly wide range of data and perspectives to design generally better digital health interventions – which may then not be perfect for any one individual.

An open question remains on the possibility of successfully formalising convergent approaches. In this case, a core issue resides in the point of conflict between design-driven data management practices and data management practices adopted in other disciplines, particularly the ones afferent to clinical research. For instance, while an exploratory approach to data collection is adopted in data-enabled design (in which data are collected and later creatively analysed), clinical guidelines for data collection are based on *apriori* estimations of usefulness, which need to be formulated in advance (Noortman et al., 2022). Furthermore, the need for integrating data created as part of the research project and data created as part of the normal care provided to patients might require the construction of complex, customised datasets and data infrastructures (Nepal et al., 2013).

As a result of these conflicts, methodological compromises become necessary for the joint data collection effort to proceed. The resolution of these interdisciplinary methodological conflicts appears to be crucial for the future of the digital health design field, in a context of increasing data-drivenness and need for continuous post-adoption evolution and re-evaluation of digital health systems. As such, we indicate the formalisation of convergent approaches as a research challenge of crucial interest for the field of digital health design. Finally, while the distinction between the four approaches strictly refers to healthcare as a digitised, highly regulated, and specialised domain, we hypothesise that advances in convergent approaches might also result in useful design insights for increasingly digital sectors such as mobility or energy production.

## 5. System-level relevance and direction for future research

The description and comparison between the four approaches to the use of data for digital health design so far has dealt with differences in their internal decision-making processes. However, a different level of analysis can be proposed, focusing on the prevalence of any of these approaches in the overall health innovation landscape at any given point and on the possible effects of this prevalence on ongoing, system-level transitions.

Currently, this landscape appears to be in a state of flux. Silent approaches appear to be still pervasive but in relative decline, due to the professionalisation of design activities and to the growing recognition of the importance of the design perspective in the (digital) health innovation arena (Tsekleves & Cooper, 2017). Conversely, overt approaches appear to be in a phase of relative maturity (Chamberlain & Craig, 2017), while data-enabled approaches keep on developing (Bogers et al., 2016, 2016, 2018, 2018; Jansen et al., 2020; Lovei et al., 2020; Van Kollenburg et al., 2018) and convergent approaches appear to be just emerging (see e.g. Sharp et al., 2016; Alwashmi et al., 2019; Pannunzio et al., 2019; 2023a). Predicting the impact of these changes at the level of the health system at large appears impossible; we expect that different “patterns of transformation” (Consoli & Mina, 2009) might emerge from the large-scale diffusion of one approach over the other, but the nature of such patterns appears arduous to predict at this stage.

Nonetheless, we must note that a common system-level dynamic of digital health innovation processes is a tendency to generate new problems on the way of solving others. Digital health innovation, in fact, is not only prone to the emergence of unintended consequences typical of the field of health information technology (Ash et al., 2004; Wachter, 2015), but also to the paradoxical effects typical of automation efforts at large (Bainbridge, 1983; Strauch, 2017), including the risk of inadvertently exacerbating existing health disparities (Berg et al., 2022). While we cannot at this stage suggest any of the four approaches to digital health design to be preferable from this point of view, we can note that convergent approaches can in principle enable innovators -as well as indipendent evaluators- to conduct iterative cycles of exploration and detection of possible unintended consequences through longitudinal, holistic system monitoring.

More at large, as previously mentioned, the diffusion of convergent approaches appears to be coherent with the long-term vision of LHSs (McGinnis et al., 2011). Indeed, the challenge of convergent approaches lies in the development of effective healthcare data systems, able to continuously inform actors from multiple disciplines using a wealth of heterogeneous, real-life data . This is, on a larger scale, precisely the challenge of LHSs (Budrionis & Bellika, 2016). In these terms, an opportunity exists for digital health design projects adopting a convergent approach to constitute a small-scale, local testing ground for the larger-scale transition towards LHSs.

Next to these system-level considerations, we believe the distinction between the four approaches to be of interest for practitioners in the field. In these terms, we intend this contribution to provide a first broad differentiation and characterisation of the approaches available to digital health designers and design managers in terms of data management, decision-making processes, and their implications.

Furthermore, we note that the notion of convergent approaches has implications from an industrial perspective, in that it could guide interdisciplinary teams to consider a broad variety of “captured” (Briard et al., 2023) data collection needs while designing digital artefacts. At the same time, the issue of convergence opens practical questions from an industrial point of view, including on the feasibility of a large-scale adoption of convergent approaches – and the organisational restructuring required to achieve such a goal. Governance structures carefully protect patient and staff data, and few existing forms of collaboration allow for the kind of longitudinal, interdisciplinary data sharing necessary for convergent design approaches. More stable forms of organisational integration between care providers and industrial partners might be necessary to achieve convergence at scale, and enable a digital health data ecosystem that is conducive to innovation, respectful of patients’ and providers’ privacy, compliant with relevant rules and regulations, and capable of performing continuous algorithmic monitoring and improvement. In this perspective, we see an opportunity for future convergent methodologies to align with and provide input for the evolving regulatory framework in the field of digital health.

Finally, we observe that collaboratively developing, applying and refining data strategies in interdisciplinary digital health design efforts constitute a chance to advance the field of systems approaches to health design, as described by Komashie et al., 2019; 2021; Ciccone et al., 2019; and Schoepen et al., 2022.

## Notes

1. Feinberg (2017), however, offers a nuanced critique of the differentiation between “using” and “designing” data, arguing that we always design data and never merely appropriate it.

2. We gratefully acknowledge an anonymous reviewer for this valuable insight.
